# Intravenous Lipid Emulsion Therapy in Drug Overdose and Poisoning: An Updated Review

**DOI:** 10.5152/eurasianjmed.2024.24510

**Published:** 2024-10-01

**Authors:** Sevdegül Bilvanisi, Müge Gülen, Mustafa Sabak, Şeniz Demiryürek, Abdullah Tuncay Demiryürek

**Affiliations:** 1 Department of Emergency Medicine, Yüzüncü Yıl University Faculty of Medicine, Van, Türkiye; 2 Clinic of Emergency Medicine, Health Sciences University Adana City Training and Research Hospital, Adana, Türkiye; 3 Department of Emergency Medicine, Gaziantep University Faculty of Medicine, Gaziantep, Türkiye; 4 Department of Physiology, Gaziantep University Faculty of Medicine, Gaziantep, Türkiye; 5 Department of Medical Pharmacology, Gaziantep University Faculty of Medicine, Gaziantep, Türkiye

**Keywords:** Antidotes, drug toxicity, emergency treatment, intravenous fat emulsion, resuscitation

## Abstract

The use of intravenous lipid emulsion (ILE) is thought to reverse the acute neurological and cardiac toxicities generated by local anesthetic and non-anesthetic drugs. The aim of this review is to provide an updated overview of ILE therapy in the management of the toxic effects of medications on humans. Indications, mechanisms of action, monitoring, dosing, lipid formulations, adverse effects, and contraindications related to ILE are highlighted. Although ILE therapy was initially utilized for local anesthetic toxicity, its use has been extended to patients with overdoses or poisoning induced by various non-local anesthetic drugs. It has been proposed that intravenous lipid droplets generate a discrete lipophilic phase in the bloodstream into which liposoluble drugs preferentially partition. This partitioning effect, known as the lipid sink phenomenon, is thought to decrease the quantity of drug content in tissues in vital organs. At the same time, other studies have also described several molecular mechanisms that may contribute to ILE efficacy. Potential adverse effects of ILE have also been identified, such as pulmonary toxicity, hypertriglyceridemia, acute pancreatitis, interference with laboratory measurements, fat overload syndrome, worsening of systemic absorption of toxin, and hepatic dysfunction. Intravenous lipid emulsion therapy is gaining wider acceptance in critical care units and emergency rooms as a possible treatment modality for liposoluble drug toxicity. Currently, recommendations on ILE administration in clinical toxicology are mainly based on published case reports and animal studies. Thus, further clinical studies are required to increase knowledge about ILE therapy.

**Main Points**

- Intravenous lipid emulsion therapy was initially utilized in the treatment of local anesthetic toxicity, but its use in acute poisoning is of growing interest.
- Given the cardiovascular benefits seen in case reports, it is now successfully used for the reversal of various non-local anesthetic drug toxicities.
- The mechanism by which lipid emulsions are so beneficial is not fully understood; therefore, more studies are required to better understand their mechanism of action, optimal dosing, other possible indications, adverse effects, and complications.

## Introduction

Lipid emulsions have been developed to supply nutritional requirements to patients unable to receive adequate enteral nutrition. The use of lipid emulsion (LE) in critically ill patients provides essential fatty acids and calories. Intravenous LE (ILE, also known as lipid resuscitation, intravenous fat emulsion, or lipid emulsion therapy) is a mixture of soybean oil, egg phospholipids, and glycerol. Intravenous lipid emulsion is also utilized as a solvent for intravenous drug delivery of liposoluble drugs such as etomidate, amphotericin, and propofol. Moreover, the potential for ILE administration to act as an antidote for the prevention of drug toxicity has been revisited within the last 3 decades.^1^ That is, LE is used to supply calories to those unable to take enteral sustenance, while ILE is the use of LE to reverse, for instance, local anesthetic toxicity. Cardiovascular failure is the most life-threatening complication of local anesthetic intravascular injections or systemic absorption during regional anesthesia. Local anesthetic systemic toxicity (LAST) is a life-endangering situation with an incidence presently estimated to be 0.03% or 0.27 episodes per 1000 peripheral nerve blocks.^2^ Intravenous lipid emulsion therapy was first applied for recovery from local anesthetic toxicity in experimental research. In 1998, Weinberg et al,^3^ documented the successful use of ILE infusion in the reversal of bupivacaine-induced toxicity in rats. Follow-up experiments in dogs confirmed the efficacy of ILE in reversing the toxic effects of bupivacaine overdose.^4^ After these reports, ILE was investigated in animal models for toxicity from a variety of liposoluble drugs. The first clinical translation of ILE administration was stated in 2 separate cases of LAST in 2006 by Rosenblatt et al^5^ and Litz et al.^6^ Furthermore, the first clinical case of ILE therapy in a patient with toxicity generated by a non-local anesthetic drug was documented in 2008. Sirianni et al^7^ reported that infusion caused the recovery of a 17-year-old female patient with severe seizures and cardiovascular depression due to toxic doses of lamotrigine and bupropion. Patients with underlying heart disease, such as baseline conduction defects and coronary artery disease, have been demonstrated to be at augmented risk for cardiotoxicity induced by local anesthetics.^1^ Intravenous lipid emulsion therapy has been effectively used to treat LAST in a broad range of patients, i.e., from neonates to the elderly.^6,8^ Patient risk groups include those at extremes of age (<6 months old and elderly), renal failure, heart failure, hepatic failure, presence of cardiac conduction defects, pregnancy, and those with certain metabolic pathway deficiencies. In these groups, the dose is reduced appropriately, and extra caution must be applied.^9^ The use of ILE as an antidote is best evaluated for the treatment of LAST. Intravenous lipid emulsion was later found to be effective in cases of intoxication with a variety of other lipophilic drugs.^2^ It has been used successfully in the overdose of several drugs. The Association of Anaesthetists of Great Britain and Ireland (AAGBI), in their 2007 guidelines for the treatment of LAST, has recommended the use of ILE therapy.^10^ Emergency physicians also recommend keeping lipid emulsions near all resuscitation rooms for toxicological emergencies. Intravenous lipid emulsion can have a considerable rescue effect, as reported in many guidelines, albeit with multiple confounding biases due to the absence of proper randomized trials.^10-12^ This paper reviews updated information and recommendations on LE therapies and provides evidence for the management of acute drug poisoning in patients. It should be pointed out that toxicities related to organophosphates, parasiticides, herbicides, pesticides, and rodenticides can also be successfully treated with ILE, but these agents are outside of the scope of this review.

## Indications

Intravenous lipid emulsion therapy is currently recommended for the treatment of LAST by professional societies, including the American Society of Regional Anesthesia and Pain Medicine (ASRA).^12^ Intravenous lipid emulsion therapy is also proposed as an adjunct to advanced cardiac life support (ACLS) protocols in suspected LAST-induced cardiac arrest, according to the American Heart Association (AHA) guidelines.^13^

The rates of LAST are still debated and may be much higher. For instance, the rate identified by Rubin et al^14^ is on the order of 2/1000. Local anesthetic systemic toxicity is sometimes refractory to ACLS measures. However, the accidental subarachnoid injection of local anesthetics can result in progressive hypotension, normovolemic shock, and fatality. The initial symptoms of LAST are secondary to central nervous system (CNS) involvement, as local anesthetics first suppress the inhibiting pathways in the brain. Cardiovascular toxicity may manifest clinically as the development of arrhythmias and myocardial depression, secondary to inhibition of sinoatrial and atrioventricular node conduction. This may manifest on an electrocardiogram as prolonged PR, widening of the QRS complex, and atrioventricular blocks of varying degrees. The patient may develop bradycardias and re-entrant tachyarrhythmias, along with life-threatening ventricular tachycardia or fibrillation. If a patient with LAST develops cardiac arrest, it may be refractory to standard resuscitative measures or general supportive treatments, more so if the involved agent is a long-acting local anesthetic like bupivacaine. The presence of cardiovascular symptoms at the time of presentation indicates severe toxicity and poor outcomes. Intravenous lipid emulsion therapy is now commonly accepted as a standard therapeutic intervention following LAST resulting from intravascular local anesthetic injections, inadvertent local anesthetic overdoses, and rapid local anesthetic absorption effects from injections in mostly vascular sites. In addition to its use during resuscitation, ILE was utilized to treat milder clinical scenarios of LAST, such as altered consciousness and cardiac dysrhythmias, without loss of pulse.^11,13^

In the setting of cardiovascular collapse, ILE is recommended with cardiopulmonary resuscitation and standard ACLS. Particularly, the mechanism of toxicity has significant implications for the management of cardiac arrest in the setting of LAST. Since standard dosing can interfere with lipid resuscitation and impair therapy, epinephrine for treating hypotension is administered to patients with LAST at lower than usual doses (boluses of <1 μg/kg). Vasopressin should be avoided, as it may further decrease tissue perfusion and cardiac output. Bradycardia and hypotension can be managed with conventional therapies.^9^ Local anesthetic drugs that can benefit from ILE administration are presented in Table 1.

## Role in Non-local Anesthetic Toxicity

Reported cases of successful resuscitation show the efficacy of ILE administration for treating non-local anesthetic toxicity across a large spectrum of drugs, including antiarrhythmics, β-blockers, tricyclic antidepressants, amphetamines, psychotropic agents, antiepileptics, and calcium channel blockers.^11^ These drugs do not have a common mechanism, site of action, clinical effects, or chemical structure, but only high lipid solubility appears to be common among them. The administration of ILE can be considered adjunctive therapy for refractory hypotension with liposoluble drug overdoses, especially if it is associated with bradycardia. Thus, ILE is a promising therapy for the treatment of drug-induced cardiogenic shock.^15^ Local and non-local anesthetic drugs, whose toxic effects can be treated with ILE as an antidote, are listed in Table 1.

### β-blockers

Intravenous lipid emulsion can have a potential role in selected cases of severe β-blocker overdose. Some reports include metoprolol,^16^ carvedilol,^16^ and propranolol overdose.^16^ There is also a report of improvement after ILE in a case of severe propranolol toxicity in a 7-month-old infant.^8^ Intravenous lipidemulsi on therapy is reasonable in β-blocker-poisoned patients who have cardiovascular collapse unresponsive to other interventions, but caution is warranted given reports of sudden asystole after LE in this situation. The optimal formulation and dose of LE for this purpose are unknown.^1,17^ Further study appears to be necessary for elucidating the effect of ILE on β-blocker toxicity.

### Calcium Channel Blockers

The first case of sustained-release verapamil-induced toxicity treated with ILE was published by Young et al^18^ in 2009. Intravenous lipid emulsion probably functions as a lipid sink that decreases free verapamil concentration in human serum and rapidly reverses depressed cardiomyocyte contractility in the continued presence of verapamil. Intravenous lipid emulsion administration has proven to be an effective and plausible therapy for verapamil and diltiazem overdoses.^17,18^ The Lipid Emulsion Workgroup’s evidence-based recommendation on the use of ILE in CCB overdose indicates that in the setting of life-threatening and non-life-threatening CCB intoxication, ILE should not be administered as first-line therapy. The use of ILE is reasonable for CCB-triggered severe cardiovascular toxicity that persists despite maximal treatment with standard resuscitative protocols and extracorporeal membrane oxygenation (ECMO) and other extracorporeal life support, or ACLS, which are not available.^11^ Despite the high number of reports, however, ILE is not yet proposed by the current guidelines for the treatment of CCB poisoning.

### Antidepressants

Intravenous lipid emulsion is indicated to be beneficial in reversing cardiovascular toxicity caused by several liposoluble antidepressants, such as quetiapine, bupropion, and amitriptyline (Table 1). Evidence-based recommendations suggest the administration of ILE for life-endangering bupropion overdose after other therapies fail.^7,11^ It was reported that the early use of LE in an overdose of amitriptyline via orogastric administration elevated plasma amitriptyline and reduced survival, implying that early administration of LE may increase the gastrointestinal absorption of highly liposoluble amitriptyline by the blood containing the LE used to perfuse the gastrointestinal mucosa.^19^ Given the currently available data, ILE is reasonable for cardiac arrest after cyclic antidepressant poisoning and for refractory hypotension or cardiac dysrhythmias if other interventions fail but should not be used as a first-line therapy and should not delay efforts to achieve sodium loading and serum alkalization in patients with cardiovascular toxicity.

### Antiarrhythmics

Intravenous lipid emulsion therapy was reportedly successful in 2 patients with severe flecainide poisoning and in propafenone poisoning.^17^ Although the safety and efficacy of ILE therapy in this setting remain undefined, it is reasonable for those who are seriously ill, deteriorating, and refractory to conventional therapy.

### Antimalarial/Antirheumatic Drugs

Intravenous lipid emulsion inhibits the late apoptosis and cardiotoxicity induced by chloroquine toxicity.^17^ As hydroxychloroquine is a lipophilic drug, hydroxychloroquine poisoning is an indication for ILE therapy.^20^ The early supplemental use of ILE might be an effective and safe treatment to inhibit cardiac toxicity after possible lethal intoxication with hydroxychloroquine.

### Other Drugs

Published reports of ILE therapy are progressively extending and have already included cases of several lipophilic drugs, such as Z-drugs,^21^ the first-generation antihistamine diphenhydramine,^17^ and caffeine.^22^ Elgazzar et al^23^ indicated that LE (SMOF lipid, 20%; a mixture of medium-chain triglycerides, soybean oil, fish oil, and olive oil) elevated the Glasgow coma scale and decreased the incidence of QT prolongation and hospital stay in acute clozapine toxicity. These data support the feasibility of LE administration in the setting of acute poisoning with clozapine. A recent case report documented that ILE was successfully utilized for treating ventricular arrhythmias induced by severe prothipendyl intoxication.^24^ It has been reported that severe dextromethorphan poisoning with serotonin syndrome was successfully treated with ILE.^25^ The ILE treatment has been shown to produce a higher level of consciousness, a decreased length of hospitalization, and a diminished rate of seizure occurrence in patients with pure tramadol poisoning.^26^

## Dosing and Administration

The most recent guidelines by the ASRA suggest dosing for a patient in cardiac arrest is a 1.5 mL/kg bolus of 20% LE rapidly over 2 to 3 minutes while continuing chest compressions, followed by a continuous infusion of 0.25 mL/kg/min.^12^ If the patient has persistent cardiovascular collapse or remains unstable, it is reasonable to repeat the bolus dose once or twice and double the infusion rate. If there is evidence of recovery, it is generally accepted to continue the infusion for at least 10 minutes after achieving cardiovascular stability. After initiating standard ACLS protocols, including ensuring adequate oxygenation and ventilation, it is recommended that ILE be given as soon as possible after signs of significant local anesthetic toxicity become manifest. Several toxicology societies and poison centers have limited the amount of ILE to an 8 mL/kg total dose and suggest several boluses rather than an infusion to limit the adverse effect.^11^ Recent guidelines indicate slower administration (over 2-3 minutes) of the initial bolus of LE.^13^ Data from several studies indicate that ILE is more effective when given early,^27^ and case reports^28^ indicate early use can prevent progression.

The precise dose of ILE for non-local anesthetics has not been studied, and it is not known if boluses or infusions are more effective. The reasonable, safe total dose is also unknown but would depend on the degree of toxicity and response to previous doses of ILE. The most common route of administration is IV, either peripherally or via central catheters. The intraosseous route was also, albeit seldom, reported as successful. Sampson and Bedy^29^ reported that ILE given via the intraosseous route successfully treated a massive verapamil overdose in a 24-year-old woman. The advantages of ILE include relatively low cost, a wide margin of safety, ease of administration, and long shelf life.^30^

Propofol also has insufficient lipid (10%) to generate an adequate plasma lipid phase without delivering an excessive volume (and, of course, a dangerous amount of propofol).^31^ If propofol is used as an ILE therapy, it would deliver a bolus dose of 12 times the clinically used dose of propofol. This would certainly exacerbate drug-induced bradycardia and hypotension. Therefore, propofol is not recommended for ILE treatment.

Due to a lack of data, the FDA has assigned LE to pregnancy category C for all trimesters. It is not known whether ILE can lead to fetal harm when given to gravid patients. Few cases of ILE resuscitation have been published in pregnant patients.^32^ Potentially, large doses can result in elevated triglyceride concentrations, and lipid globules may occlude the placental vasculature. The risk of potential toxicity should be weighed against the potential benefit to the pregnant woman and fetus. Recent data suggest that pregnant women who received ILE for LAST survived, and none of them had sustained lasting neurological or cardiovascular damage related to LAST.^9^ Additionally, no side effects or adverse events following ILE administration were reported in neonates or mothers.^9^ There is no reported risk of ILE in breastfeeding infants.

## Monitoring

Monitoring should focus on the physiological toxic effects one is trying to reverse, e.g., cardiovascular and CNS instability, as well as possible signs of adverse effects of ILE. Because of the potential for allergic reactions, monitoring for symptoms and signs such as fever, cyanosis, or dyspnea is very important. The patients should be carefully monitored for the development of any allergic reactions, especially during the initial infusion. Lipemia is a goal of ILE and is expected; patients need evaluation for symptoms of pancreatitis and hypertriglyceridemia. Close monitoring is also required for triglyceride levels, particularly when doses are adjusted. Bilirubin concentrations, liver enzyme tests, and lipase levels are necessary if there is a suspicion of acute cholecystitis or pancreatitis.^2,11,30^

## Mechanism of Action

The exact mechanism of action of ILE is not completely identified. In the first experimental study, Weinberg et al^3^ proposed different theories to clarify the quick reversal of severe bupivacaine toxicity. These included a metabolic effect, inhibition of nitric oxide production, the possibility that the newly created lipid plasma phase functioned as a lipid sink, and the redistribution of the drug. Lipids may also directly bind to ion channels.^1^ Collectively, the proposed mechanisms of action of ILE include lipid sink theory, modulation of cellular signaling and metabolic processing, and activation of ion channels.

### Lipid Sink Theory

The lipid sink is a mechanism of action in which liposoluble local anesthetics are absorbed into the lipid phase of plasma from tissues affected by drug-induced toxicity. Thus, the entrapment of drugs in the serum lipid phase could decrease the amount of drug at the site of toxicity. In other words, the lipid phase creates a concentration gradient for the drug in the tissues, sequestering the drug from the tissue into the plasma. Intravenous lipid emulsion may transport any liposoluble drug from well-perfused organs (the kidney, heart, and brain) to detoxification organs such as the liver. This phenomenon is known as the lipid shuttle.^1^

### Modulation of Cellular Signaling and Metabolic Processing

Intravenous lipid emulsion induces alleviation of mitochondrial dysfunction, suppression of nitric oxide release, generation of a positive inotropic effect, supplying fatty acids, and glycogen synthase kinase-3β phosphorylation linked with attenuation of mitochondrial permeability transition pore (mPTP) opening.^1^ It is known that mitochondrial function is closely associated with energy metabolism. The mPTP opening is the critical stage of programmed cell death, and ILE can restore the function of mitochondria by suppressing the mPTP opening. Inhibition of mPTP opening has also been described as a potential mechanism for the successful rescue of bupivacaine-stimulated cardiotoxicity by ILE. Intravenous lipid emulsion also inhibits apoptosis stimulated by toxic doses of bupivacaine through amelioration of mitochondrial dysfunction and elevated oxidative stress induced by bupivacaine in rat cardiomyocytes.^33^

Lipid emulsion and increased free-fatty acids produce vasoconstriction, probably by interacting with nitric oxide signaling or by modifying adrenergic sensitivity.^34,35^ ILE may reverse the severe vasodilation induced by LAST via inhibition of endothelial nitric oxide synthase (eNOS). However, there is no doubt that cardiovascular function does not improve until the cardiac concentration of a local anesthetic drug falls below sodium channel blocking thresholds.

Bupivacaine suppresses respiratory chain complexes 1 and 3, causing augmented reactive oxygen species (ROS) generation.^36^ There is evidence showing that ILE has free radical-scavenging effects at high concentrations.^37^ Specifically, ILE can have antioxidant activity against hydroxyl radicals.^37^

Intravenous lipid emulsion therapy can also reverse the CNS symptoms of LAST. It has been reported that bupivacaine particularly damages the mitochondrial function of astrocytes, thereby reducing glutamate uptake and indirectly increasing the calcium signal transduction in neurons triggered by glutamate.^38^ Intravenous lipid emulsion can prevent bupivacaine-induced damage to the CNS.

Postconditioning myocardial protection may also occur with ILE therapy.^39^ Lipid exerts postconditioning effects via stimulation of prosurvival kinases, along with vasoconstrictive and cardiotonic effects. These beneficial effects protect tissue from ischemic injury and enhance tissue perfusion during recovery from toxicity.

### Activation of Ion Channels

**Figure 1.**
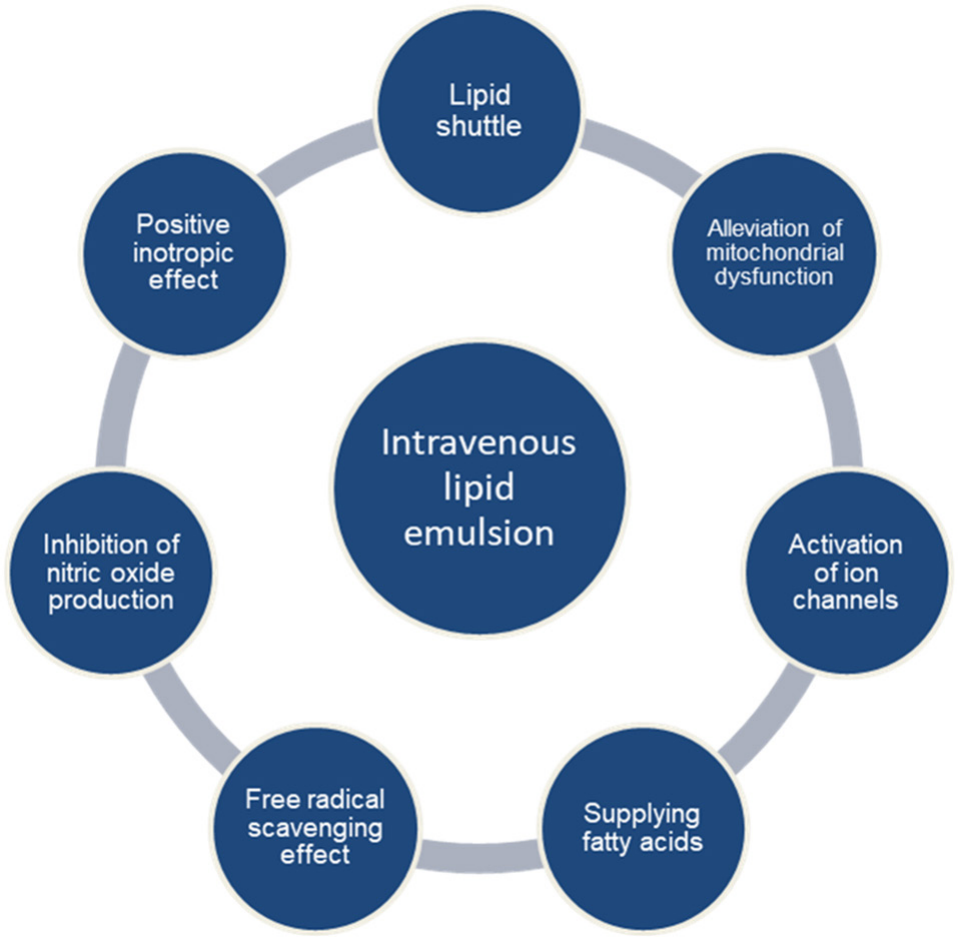
Schematic representation of suggested mechanisms of action for intravenous lipid emulsion.

Intravenous lipid emulsion infusion might directly elevate intracellular calcium levels in cardiomyocytes and cause a direct positive inotropic effect. Although intravenous injection of a 20% fat emulsion alone does not initiate arrhythmias, the synergistic arrhythmogenic actions of free fatty acids and ouabain can be described by the suppressing effect of both agents on membrane Na^+^-K^+^-ATPase.^40^ Proposed mechanisms of ILE action are summarized in Fig. 1.

## Lipid Preparations and Formulations

Although Intralipid^®^ is the most prevalent commercial medicinal product utilized in documented resuscitations, there are various ILE formulations using numerous lipid sources and concentrations. There are different commercially available 20% ILE preparations. Soybean oil appears to be the predominant ILE in clinical use and experimental research studies for the treatment of LAST. American Society of Regional Anesthesia and Pain Medicine does not recommend a specific brand of LE. Intralipid^®^ is the recommended LE.^12^ The content of Intralipid^®^ consists of soybean oil (20%), egg yolk phospholipids (1.2%), glycerin (2.25%), and water. The 20% Liposyn^®^ and 20% Medialipid^®^ can be used.^17^ An alternate formulation, Liposyn II^®^ 20% injection, also comes with safflower oil (10%), soybean oil (10%), and egg phosphatides (1.2%). Others on the market consist of 50% long-chain fatty acids from soybean oil and 50% medium-chain fatty acids from coconut oil. Lipid emulsions incorporating fish and olive oils, which are commonly prescribed as parenteral nutrition, are used less frequently as antidotes.^1,17^ LE is available in parenteral formulations of 5%, 10%, 20%, and 30% solutions. The 20% solution is the formulation that is recommended and the one used most often. The 30% solution is a pharmacy bulk admixture and is used to prepare dilute concentrations. The 30% solution should be diluted before use and has not been administered clinically for drug toxicity. Most case reports support the ILE treatment for local anesthetic toxicity using branded Intralipid^®^ or standard long-chain triglyceride mixtures.

Controversy exists as to whether or not lipid emulsions containing both long- and medium-chain triglyceride mixtures are more effective at partitioning drugs than long-chain triglycerides.^41^ In human serum, a mixture of medium- and long-chain triglycerides increased the extraction of bupivacaine, ropivacaine, and mepivacaine compared with long-chain triglyceride mixtures alone.^41^ Thus, further research is required to identify the appropriate dosing formulation with triglycerides or with novel non-triglyceride formulations.

## Adverse Effects and Contraindications

The reported rate of adverse effects of ILE administration is very low. Because few publications report adverse events following antidotal use of ILE, the true incidence remains largely unexplored.^42^

### As a Parenteral Nutrition Intravenous Lipid Emulsion

It is likely that for most of them, either large or repeated dose administration as a parenteral nutrition is a necessary condition for adverse effects to occur.^42^ Anecdotal cases of insulin resistance, allergic reactions to soybeans, or pancreatitis have been reported.^42^ Hypertriglyceridemia and pancreatitis can be observed when ILE is used for parenteral nutrition. Pulmonary toxicity is noted when ILE is administered as a source of parenteral nutrition.^43^ ILE may occlude the pulmonary vasculature with microfat emboli. Nevertheless, it should be pointed out that most complications are linked with large doses of LE, generally beyond the recommended dosing.

### Potential Complications of Intravenous Lipid Emulsion 

Potential complications of ILE include ventilation/perfusion mismatch, ARDS, cardiac arrest, hypersensitivity, venous thromboembolism, hypertriglyceridemia, acute kidney injury, fat embolism, pancreatitis, fat overload syndrome, increased susceptibility to infection, local vein irritation, extracorporeal circulation machine circuit obstruction, electrolyte disturbances, and allergic reactions.^42^ Cases of acute lung injury have been reported after the initiation of ILE, which may lead to acute hypoxia. Hepatic dysfunction has recently been reported in patients receiving ILE.^44^ Therefore, liver function should also be monitored. Hyperamylasemia and pancreatitis were reported in 2 cases of ILE use in the management of drug toxicity.^13^ The development of acute pancreatitis seems to be dose-related, and the risk is highest in those patients who receive several doses or a prolonged lipid infusion. The precise mechanism of ILE-induced pancreatitis is unknown, but it may be the result of the bulky concentration of triglycerides forming large lipid droplets that obstruct the small vessels of the pancreas, causing ischemia. Lipase then degrades the triglycerides, releasing cytotoxic free fatty acids. It is recommended that if the patient develops hypertriglyceridemia (triglyceride levels >400 mg/dL), then the dose of ILE should be reduced, but if the serum triglyceride levels exceed 1000 mg/dL, then ILE should be completely stopped. Large doses or rapid infusions of ILE have the potential to produce a fat overload syndrome, which is defined as hepatomegaly, headaches, fat infiltration, hyperlipidemia, jaundice, fever, splenomegaly, thrombocytopenia, seizures, hemolytic anemia, coagulopathy, leukopenia, and coma.^45^ Fat accumulation may cause steatosis, cholestasis, and gallbladder sludge. Due to the rapid redistribution of most local anesthetics, prolonged ILE infusions should not be needed. However, many other liposoluble drugs have a long duration of toxicity, and repeated and prolonged ILE infusions, if given, increase the risk of fat overload syndrome. Particularly when administered early in the clinical course of an oral overdose, ILE can have the potential to enhance gastrointestinal absorption or facilitate the distribution of liposoluble drugs, resulting in increased toxicity. In an orogastric model of amitriptyline overdose, ILE increased amitriptyline concentrations and resultant toxicity.^19^ Gastric decontamination should be performed under these circumstances.

### Changes Clinical Laboratory Parameters after Intravenous Lipid Emulsion 

The use of high-dose lipids in the form of ILE obviously leads to hyperlipidemia, which may make the blood sample analysis uninterpretable.^46^ Intravenous lipid emulsion variably alters analytical test results and results in erroneous measurements, no significant effect, or the inability to perform a laboratory test. In the setting of ILE, glucose measurement by the colorimetric method did not accurately report hypoglycemia. Sodium, potassium, calcium, chloride, bicarbonate, troponin-I, or urea assays had the least interference. Magnesium and albumin assays demonstrated significant interference. Total protein, lipase, amylase, phosphate, creatinine, alanine aminotransferase, creatine kinase, and bilirubin became unmeasurable.^46^

### Contraindications of Intravenous Lipid Emulsion 

ILE administration is contraindicated for patients with severe egg allergies. However, the risk of allergy needs to be measured against the benefit of ILE therapy. Severe sepsis has been described as a contraindication to ILE use.^42^ Relative contraindications for ILE include pancreatitis, pulmonary disease, and fat metabolism disorders. Caution should also be taken when administering to a patient with lipid storage disorders and impaired lipid metabolism. Despite many adverse effects being attributed to ILE therapy, more in-depth results are required to clarify the risk of complications from ILE treatment in patients with acute toxicity. The reported adverse effects of ILE use are summarized in Table 2.

## Intravenous Lipid Emulsion and Drug Interactions

ILE administration can interfere with therapeutic drug monitoring. The effect of ILE on toxic drug concentration assays is largely unknown. Ideally, blood specimens should be collected in advance before ILE administration, and then analytic testing should be performed. Interferences can be decreased by low-speed centrifugation.^46^ Moreover, it should also be kept in mind that this interference may persist even up to 24 hours after stopping the ILE.^46^ Intravenous lipid emulsion also has the potential to interact with other essential antidotes, especially epinephrine and vasopressin.^47^ Intravenous lipid emulsion was also utilized with several other drugs during resuscitation for toxicity, including magnesium sulfate, atropine, naloxone, sodium bicarbonate, calcium chloride, and metaraminol.^48^

## Delayed or Recurring Toxicity of Intravenous Lipid Emulsion Use

Several reports of delayed or recurring toxicity have been reported following ILE. These effects may appear even after 24 hours of stopping lipid infusion and may manifest as worsening of the sensorium, the development of seizures, arrhythmias, or even cardiac arrest. Hence, every patient should be monitored for the development of such complications even after stopping ILE.^30,42^

## Extracorporeal Circulation Machine Circuit Obstruction

Clinical and in vitro experimental studies suggest that the combined use of ECMO and ILE can be closely connected with fat deposition in the veno-arterial ECMO circuits and augmented blood clot formation.^49,50^

## Conclusion

The use of ILE in LAST and toxidromes from drugs other than local anesthetics has been extensively documented. Presently, any signs or symptoms of LAST should be treated with ILE. Intravenous lipid emulsion is usually considered easy to obtain and economical for the first-line preparation for the LAST treatment. Lipid emulsion use for other drugs is only reasonable when severe toxicity resulting from lipid-soluble drugs persists despite maximum treatment with standard resuscitation measures. Thus, ILE has become a first-line treatment for LAST, but its use for non-LAST toxicity should be reserved for those patients with life-endangering toxicity that is unresponsive to standard therapies. Although ILE has achieved remarkable success in the treatment of LAST, contraindications and adverse reactions are also present; therefore, a high degree of attention is required during ILE administration.

This comprehensive review has identified recently published clinical studies and case reports indicating successful ILE use for the reversal of various drug toxicities. As the number of patients resuscitated with ILE therapy for drug toxicity has progressively increased, there has been greater awareness of the adverse effects of ILE. Despite the growing awareness of ILE therapy, more clinical studies are required to better understand its mechanism of action, optimal dosing, other possible indications, and accompanying complications.

## Data Availability Statement

The data that support the findings of this study are available on request from the corresponding author.

## Figures and Tables

**Table 1.** Local and Non-local Anesthetic Drug Intoxicants for Which Reports Indicate Possible Benefit with Intravenous Lipid Emulsion

Local and Non-local Anesthetic drugs Toxic Effects can be Treated with Intravenous Lipid Emulsion Administration				
**Local anesthetics**	**Antidepressants**	**Antipsychotics**	**Barbiturates**	**SNRI**
Bupivacaine	Amitriptyline	Acepromazine	Pentobarbital	Atomoxetine
Cocaine	Amoxapine	Chlorpromazine	Phenobarbital	**Antihypertensives **
Levobupivacaine	Bupropion	Clozapine	Thiopental	Clonidine
Lidocaine	Citalopram	Haloperidol	**Amphetamines**	Doxazosin
Mepivacaine	Clomipramine	Olanzapine	Amphetamine	**Antimalarial **
Prilocaine	Desvenlafaxine	Prothipendyl	Methamphetamine	**Antirheumatic**
Ropivacaine	Dosulepin	Quetiapine	**Antiarrhythmics**	**drugs**
**β-blockers**	Dothiepin	Trazodone	Amiodarone	Chloroquine
Atenolol	Doxepin	**CCBs**	Flecainide	Hydroxychloroquine
Bisoprolol	Escitalopram	Amlodipine	Propafenone	**Skeletal muscle relaxants**
Carvedilol	Fluoxetine	Diltiazem	**Antihistamines**	Baclofen
Labetalol	Imipramine	Nifedipine	Diphenhydramine	Cyclobenzaprine
Metoprolol	Mirtazapine	Verapamil	Hydroxyzine	**Z-drugs**
Nebivolol	Quetiapine	**Antiepileptics**	**Opioid analgesics**	Zolpidem
Propranolol	Sertraline	Carbamazepine	Tramadol	Zopiclone
**Antidiabetic**	Trazodone	Lamotrigine	**Anthelmintic**	**Antitussive **
Metformin	Venlafaxine	Phenytoin	Ivermectin	Dextromethorphan
**Bronchodilator **	**Antidiarrheal**	**Benzodiazepines**	**CNS stimulant**	
Theophylline	Loperamide	Alprazolam	Caffeine	

CCBs, calcium channel blockers; CNS, central nervous system; SNRI, selective norepinephrine reuptake inhibitor.

**Table 2.** Reported Adverse Effects of Intravenous Lipid Emulsion Administration

Adverse Effects of Intravenous Lipid Emulsion Therapy	
Acute kidney injury	Hypersensitivity and allergic reactions
Acute pancreatitis	Hypertriglyceridemia
Cardiovascular effects	Increased susceptibility to infection
Asystole	Interference with clinical laboratory measurements
Cardiac arrest	Priapism
Local vein irritation	Pulmonary adverse effects
Fat embolism disorder	Acute lung injury
Fat overload syndrome	Adult respiratory distress syndrome
Hematologic effects	Ventilation/perfusion mismatch
Hemolytic anemia	Electrolyte disturbances
Venous thromboembolism	Extracorporeal circulation machine circuit obstruction
Hepatic dysfunction	

## References

[1] Ok SH Hong JM Lee SH Sohn JT . Lipid emulsion for treating local anesthetic systemic toxicity. Int J Med Sci. 2018;15(7):713 722. (10.7150/ijms.22643) 29910676 PMC6001420

[2] El-Boghdadly K Pawa A Chin KJ . Local anesthetic systemic toxicity: current perspectives. Local Reg Anesth. 2018;11:35 44. (10.2147/LRA.S154512) 30122981 PMC6087022

[3] Weinberg GL VadeBoncouer T Ramaraju GA Garcia-Amaro MF Cwik MJ . Pretreatment or resuscitation with a lipid infusion shifts the dose-response to bupivacaine-induced asystole in rats. Anesthesiology. 1998;88(4):1071 1075. (10.1097/00000542-199804000-00028) 9579517

[4] Weinberg G Ripper R Feinstein DL Hoffman W . Lipid emulsion infusion rescues dogs from bupivacaine-induced cardiac toxicity. Reg Anesth Pain Med. 2003;28(3):198 202. (10.1053/rapm.2003.50041) 12772136

[5] Rosenblatt MA Abel M Fischer GW Itzkovich CJ Eisenkraft JB . Successful use of a 20% lipid emulsion to resuscitate a patient after a presumed bupivacaine-related cardiac arrest. Anesthesiology. 2006;105(1):217 218. (10.1097/00000542-200607000-00033) 16810015

[6] Litz RJ Popp M Stehr SN Koch T . Successful resuscitation of a patient with ropivacaine-induced asystole after axillary plexus block using lipid infusion. Anaesthesia. 2006;61(8):800 801. (10.1111/j.1365-2044.2006.04740.x) 16867094

[7] Sirianni AJ Osterhoudt KC Calello DP , et al. Use of lipid emulsion in the resuscitation of a patient with prolonged cardiovascular collapse after overdose of bupropion and lamotrigine. Ann Emerg Med. 2008;51(4):412-415.e1. (10.1016/j.annemergmed.2007.06.004) 17766009

[8] Thompson AM Franco Palacios CR Henriksen MN . Intravenous lipid emulsion and high-dose insulin as adjunctive therapy for propranolol toxicity in a pediatric patient. Am J Health Syst Pharm. 2016;73(12):880 885. (10.2146/ajhp150594) 27261238

[9] Tsuji M Nii M Furuta M , et al. Intravenous lipid emulsion for local anaesthetic systemic toxicity in pregnant women: a scoping review. BMC Pregnancy Childbirth. 2024;24(1):138. (10.1186/s12884-024-06309-1) 38355477 PMC10865663

[10] Picard J Ward SC Zumpe R Meek T Barlow J Harrop-Griffiths W . Guidelines and the adoption of ‘lipid rescue’ therapy for local anaesthetic toxicity. Anaesthesia. 2009;64(2):122 125. (10.1111/j.1365-2044.2008.05816.x) 19143686

[11] Gosselin S Hoegberg LCG Hoffman RS , et al. Evidence-based recommendations on the use of intravenous lipid emulsion therapy in poisoning. Clin Toxicol (Phila). 2016;54(10):899 923. (10.1080/15563650.2016.1214275) 27608281

[12] Neal JM Neal EJ Weinberg GL . American Society of regional anesthesia and pain medicine local anesthetic systemic toxicity checklist: 2020 version. Reg Anesth Pain Med. 2021;46(1):81 82. (10.1136/rapm-2020-101986) 33148630

[13] Lavonas EJ Akpunonu PD Arens AM , et al. 2023 American Heart Association focused update on the management of patients with cardiac arrest or life-threatening toxicity due to poisoning: an update to the American Heart Association guidelines for cardiopulmonary resuscitation and emergency cardiovascular care. Circulation. 2023;148(16):e149 e184. (10.1161/CIR.0000000000001161) 37721023

[14] Rubin DS Matsumoto MM Weinberg G Roth S . Local anesthetic systemic toxicity in total joint arthroplasty: incidence and risk factors in the United States from the national inpatient sample 1998-2013. Reg Anesth Pain Med. 2018;43(2):131 137. (10.1097/AAP.0000000000000684) 29280923 PMC5777869

[15] Herrman NWC Kalisieski MJ Fung C . Bupropion overdose complicated by cardiogenic shock requiring vasopressor support and lipid emulsion therapy. J Emerg Med. 2020;58(2):e47 e50. (10.1016/j.jemermed.2019.11.029) 31911020

[16] Sebe A Dişel NR Açıkalın Akpınar A Karakoç E . Role of intravenous lipid emulsions in the management of calcium channel blocker and β-blocker overdose: 3 years experience of a university hospital. Postgrad Med. 2015;127(2):119 124. (10.1080/00325481.2015.1012480) 25684131

[17] García-Ramos S Fernandez I Zaballos M . Lipid emulsions in the treatment of intoxications by local anesthesics and other drugs. Review of mechanisms of action and recommendations for use. Rev Esp Anestesiol Reanim (Engl Ed). 2022;69(7):421 432. (10.1016/j.redare.2021.03.018) 35871141

[18] Young AC Velez LI Kleinschmidt KC . Intravenous fat emulsion therapy for intentional sustained-release verapamil overdose. Resuscitation. 2009;80(5):591 593. (10.1016/j.resuscitation.2009.01.023) 19282085

[19] Perichon D Turfus S Gerostamoulos D Graudins A . An assessment of the in vivo effects of intravenous lipid emulsion on blood drug concentration and haemodynamics following oro-gastric amitriptyline overdose. Clin Toxicol (Phila). 2013;51(4):208 215. (10.3109/15563650.2013.778994) 23530458

[20] Noda K Akioka S Kubo H Hosoi H . Detoxification with intravenous lipid emulsion for fatal hydroxychloroquine poisoning. Mod Rheumatol. 2021;31(3):772 774. (10.1080/14397595.2020.1812869) 32815437

[21] Hillyard SG Barrera-Groba C Tighe R . Intralipid reverses coma associated with zopiclone and venlafaxine overdose. Eur J Anaesthesiol. 2010;27(6):582 583. (10.1097/EJA.0b013e3283357049) 20090535

[22] Muraro L Longo L Geraldini F Bortot A Paoli A Boscolo A . Intralipid in acute caffeine intoxication: a case report. J Anesth. 2016;30(5):895 899. (10.1007/s00540-016-2198-x) 27272169

[23] Elgazzar FM Elgohary MS Basiouny SM Lashin HI . Intravenous lipid emulsion as an adjuvant therapy of acute clozapine poisoning. Hum Exp Toxicol. 2021;40(7):1053 1063. (10.1177/0960327120983873) 33401984

[24] Meunier A Goffin P Deville M Demaret P . Intravenous lipid emulsion for a life-threatening prothipendyl intoxication. BMJ Case Rep. 2024;17(1):e256417. (10.1136/bcr-2023-256417) PMC10806955 38199666

[25] Kuwana T Kinoshita K Mizuochi M , et al. Administration of intravenous lipid emulsion for dextromethorphan poisoning with serotonin syndrome: a case report. J Pers Med. 2024;14(3):242. (10.3390/jpm14030242) 38540984 PMC10971483

[26] Behnoush AH Alizadeh N Emami M Bazmi E Alimohamadi Y Behnoush B . Effects of intravenous lipid emulsion administration in acute tramadol poisoning: a randomized controlled trial. J Emerg Med. 2024;66(2):154 162. (10.1016/j.jemermed.2023.11.004) 38309983

[27] Dureau P Charbit B Nicolas N Benhamou D Mazoit JX . Effect of Intralipid® on the dose of ropivacaine or levobupivacaine tolerated by volunteers: a clinical and pharmacokinetic study. Anesthesiology. 2016;125(3):474 483. (10.1097/ALN.0000000000001230) 27404223

[28] McCutchen T Gerancher JC . Early intralipid therapy may have prevented bupivacaine-associated cardiac arrest. Reg Anesth Pain Med. 2008;33(2):178 180. (10.1016/j.rapm.2007.09.006) 18299100

[29] Sampson CS Bedy SM . Lipid emulsion therapy given intraosseously in massive verapamil overdose. Am J Emerg Med. 2015;33(12):1844.e1. (10.1016/j.ajem.2015.04.061) 26003744

[30] Karcioglu O . Use of lipid emulsion therapy in local anesthetic overdose. Saudi Med J. 2017;38(10):985 993. (10.15537/smj.2017.10.20525) 28917061 PMC5694647

[31] Mayr VD Raedler C Wenzel V Lindner KH Strohmenger HU . Lipid, not propofol, treats bupivacaine overdose. Anesth Analg. 2004;99(6):1876. (10.1213/01.ANE.0000138550.57760.22) 15562099

[32] Dun-Chi Lin J Sivanesan E Horlocker TT Missair A . Two for one: a case report of intravenous lipid emulsion to treat local anesthetic systemic toxicity in term pregnancy. A Case Rep. 2017;8(9):235 237. (10.1213/XAA.0000000000000477) PMC6484440 28099175

[33] Chen Z Jin Z Xia Y , et al. The protective effect of lipid emulsion in preventing bupivacaine-induced mitochondrial injury and apoptosis of H9C2 cardiomyocytes. Drug Deliv. 2017;24(1):430 436. (10.1080/10717544.2016.1261379) 28165812 PMC8241039

[34] Steinberg HO Paradisi G Hook G Crowder K Cronin J Baron AD . Free fatty acid elevation impairs insulin-mediated vasodilation and nitric oxide production. Diabetes. 2000;49(7):1231 1238. (10.2337/diabetes.49.7.1231) 10909983

[35] Haastrup AT Stepniakowski KT Goodfriend TL Egan BM . Intralipid enhances alpha1-adrenergic receptor mediated pressor sensitivity. Hypertension. 1998;32(4):693 698. (10.1161/01.hyp.32.4.693) 9774365

[36] Cela O Piccoli C Scrima R , et al. Bupivacaine uncouples the mitochondrial oxidative phosphorylation, inhibits respiratory chain complexes I and III and enhances ROS production: results of a study on cell cultures. Mitochondrion. 2010;10(5):487 496. (10.1016/j.mito.2010.05.005) 20546950

[37] Demiryürek AT Cinel I Kahraman S , et al. Propofol and intralipid interact with reactive oxygen species: a chemiluminescence study. Br J Anaesth. 1998;80(5):649 654. (10.1093/bja/80.5.649) 9691871

[38] Xing Y Zhang N Zhang W Ren LM . Bupivacaine indirectly potentiates glutamate-induced intracellular calcium signaling in rat hippocampal neurons by impairing mitochondrial function in cocultured astrocytes. Anesthesiology. 2018;128(3):539 554. (10.1097/ALN.0000000000002003) 29232206

[39] Rahman S Li J Bopassa JC , et al. Phosphorylation of GSK-3β mediates intralipid-induced cardioprotection against ischemia/reperfusion injury. Anesthesiology. 2011;115(2):242 253. (10.1097/ALN.0b013e318223b8b9) 21691195 PMC3322241

[40] Sánchez-Serrano D Alvarez JL García-Barreto D . Enhancement of ouabain arrhythmias by fatty acids. J Cardiovasc Pharmacol. 1980;2(3):331 335. (10.1097/00005344-198005000-00010) 6156330

[41] Ruan W French D Wong A Drasner K Wu AHB . A mixed (long- and medium-chain) triglyceride lipid emulsion extracts local anesthetic from human serum in vitro more effectively than a long-chain emulsion. Anesthesiology. 2012;116(2):334 339. (10.1097/ALN.0b013e318242a5f1) 22273855

[42] Hayes BD Gosselin S Calello DP , et al. Systematic review of clinical adverse events reported after acute intravenous lipid emulsion administration. Clin Toxicol (Phila). 2016;54(5):365 404. (10.3109/15563650.2016.1151528) 27035513

[43] Venus B Prager R Patel CB Sandoval E Sloan P Smith RA . Cardiopulmonary effects of intralipid infusion in critically ill patients. Crit Care Med. 1988;16(6):587 590. (10.1097/00003246-198806000-00004) 3371022

[44] Miles JM . Hepatic dysfunction in patients receiving intravenous lipid emulsions. Curr Opin Clin Nutr Metab Care. 2023;26(3):278 283. (10.1097/MCO.0000000000000924) 36943142

[45] Hojsak I Kolaček S . Fat overload syndrome after the rapid infusion of SMOFlipid emulsion. JPEN J Parenter Enter Nutr. 2014;38(1):119 121. (10.1177/0148607113482001) 23520135

[46] Grunbaum AM Gilfix BM Hoffman RS , et al. Review of the effect of intravenous lipid emulsion on laboratory analyses. Clin Toxicol (Phila). 2016;54(2):92 102. (10.3109/15563650.2015.1115515) 26623668

[47] Hiller DB Gregorio GD Ripper R , et al. Epinephrine impairs lipid resuscitation from bupivacaine overdose: a threshold effect. Anesthesiology. 2009;111(3):498 505. (10.1097/ALN.0b013e3181afde0a) 19704251

[48] Cave G Harvey M Graudins A . Intravenous lipid emulsion as antidote: a summary of published human experience. Emerg Med Australas. 2011;23(2):123 141. (10.1111/j.1742-6723.2011.01398.x) 21489160

[49] Buck ML Ksenich RA Wooldridge P . Effect of infusing fat emulsion into extracorporeal membrane oxygenation circuits. Pharmacotherapy. 1997;17(6):1292 1295. (10.1002/j.1875-9114.1997.tb03094.x) 9399613

[50] Sin JH Tom A Toyoda A Roy N Hayes BD . High-dose intravenous lipid emulsion affecting successful initiation of continuous venovenous hemofiltration and extracorporeal membrane oxygenation. Clin Toxicol (Phila). 2018;56(2):149 150. (10.1080/15563650.2017.1341633) 28681624

